# Three-Dimensional Speckle Tracking Echocardiography for Detection of Acute Coronary Occlusions in Non-ST-Elevation Acute Coronary Syndrome Patients

**DOI:** 10.3390/diagnostics15151864

**Published:** 2025-07-25

**Authors:** Thomas M. Stokke, Kristina H. Haugaa, Kristoffer Russell, Thor Edvardsen, Sebastian I. Sarvari

**Affiliations:** 1 ProCardio Center for Innovation, Department of Cardiology, Oslo University Hospital, Rikshospitalet, P.O. Box 4950 Nydalen, 0424 Oslo, Norway; thomas.muri.stokke@gmail.com (T.M.S.); k.i.h.h.haugaa@medisin.uio.no (K.H.H.); krruss@ous-hf.no (K.R.); sebims1@hotmail.com (S.I.S.); 2 Institute for Surgical Research, Oslo University Hospital, Rikshospitalet, P.O. Box 4950 Nydalen, 0424 Oslo, Norway; 3 Institute for Clinical Medicine, University of Oslo, P.O. Box 1171 Blindern, 0318 Oslo, Norway

**Keywords:** three-dimensional echocardiography, two-dimensional echocardiography, global longitudinal strain, global circumferential strain, non-ST-elevation acute coronary syndrome, coronary occlusion

## Abstract

**Objectives**: This study aimed to evaluate the ability of three-dimensional (3D) speckle tracking echocardiography (STE) to detect acute coronary occlusions in patients with non-ST-segment elevation acute coronary syndrome (NSTE-ACS) and its potential diagnostic advantage over two-dimensional (2D) STE. **Methods**: Fifty-six patients with NSTE-ACS (mean age 64 ± 11 years; 80% male) underwent 2D and 3D transthoracic echocardiography prior to coronary angiography. Global longitudinal strain (GLS), global circumferential strain (GCS), and 3D ejection fraction (EF) were analyzed. Acute coronary occlusion was defined as TIMI flow 0–1 in the presumed culprit artery. **Results**: Acute coronary occlusion was present in 16 patients (29%). Patients with occlusion had significantly more impaired strain compared to those without: 3D GLS (−12.5 ± 2.7% vs. −15.5 ± 2.1%, *p* < 0.001), 2D GLS (−12.6 ± 2.8% vs. −15.6 ± 2.0%, *p* < 0.001), 3D GCS (−24.8 ± 4.4% vs. −27.8 ± 4.3%, *p* = 0.02), and 2D GCS (−18.1 ± 5.5% vs. −22.9 ± 4.7%, *p* = 0.002). In contrast, 3D EF did not differ significantly between groups (52.5 ± 4.7% vs. 54.7 ± 5.7%, *p* = 0.16). Receiver operating characteristic analysis showed that 3D and 2D GLS had the highest diagnostic performance (AUCs 0.81 and 0.78), while 3D EF had the lowest (AUC 0.61). Feasibility was lower for 3D STE (86%) than for 2D longitudinal strain (95%, *p* = 0.03). **Conclusions**: Both 3D and 2D GLS showed higher diagnostic accuracy than 3D EF in identifying acute coronary occlusion in NSTE-ACS patients. While 3D STE enables simultaneous assessment of multiple parameters, it did not offer incremental diagnostic value over 2D STE and had lower feasibility.

## 1. Introduction

Acute coronary syndromes (ACS) are conventionally classified by ECG findings into ST-elevation myocardial infarction (STEMI) and non–ST-elevation ACS (NSTE-ACS), which includes non–ST-elevation myocardial infarction (NSTEMI) and unstable angina (UA) [1]. While STEMI typically results from complete coronary occlusion requiring urgent intervention, NSTE-ACS has traditionally been associated with subtotal obstruction and subendocardial ischemia and is generally managed with a less immediate invasive approach [2].

However, acute coronary occlusion is reported in 25–30% of NSTEMI patients undergoing coronary angiography. These patients are at increased risk and may benefit from reperfusion strategies similar to those used in STEMI [3,4].

Acute occlusion in the absence of ST segment elevation may be explained by various mechanisms, including the location and size of the affected artery, pre-existing collateral circulation, intermittent thrombotic occlusion with spontaneous reperfusion, or early resolution of ST segment elevation before presentation [2].

Identifying this high-risk subgroup remains challenging, as ECG findings in NSTE-ACS are frequently nonspecific or absent. In this context, non-invasive imaging techniques may help bridge the diagnostic gap. Speckle-tracking echocardiography (STE) enables a quantitative assessment of myocardial deformation and can detect subtle regional left ventricular (LV) dysfunction not evident on visual inspection [5]. Two-dimensional (2D) STE has demonstrated diagnostic value in identifying both significant coronary artery disease and acute occlusion [6,7,8]. Nevertheless, 2D STE requires acquisition from multiple apical views and is limited by geometric assumptions and tracking challenges [9].

Three-dimensional (3D) STE offers a potential advantage by capturing the entire left ventricle in a single full-volume acquisition, enabling simultaneous analysis of multiple strain parameters [10]. Although it requires higher image quality and more advanced equipment, 3D STE simplifies the workflow and may improve feasibility in acute settings [11]. To date, few studies have evaluated its diagnostic utility in NSTE-ACS [12,13,14].

This study aimed to assess the ability of 3D STE to identify acute coronary occlusion in patients with NSTE-ACS and to determine whether it provides additional diagnostic value compared to 2D STE.

## 2. Materials and Methods

### 2.1. Patients and Data Collection

This prospective observational study was conducted at a single tertiary coronary care center (Oslo University Hospital, Rikshospitalet, Oslo, Norway). All patients were initially admitted acutely to local hospitals with suspected NSTE-ACS and subsequently transferred to the tertiary center for coronary angiography. Due to logistical constraints, patient enrollment occurred intermittently, constituting a convenience sampling approach rather than strict consecutive inclusion.

Inclusion criteria were age >18 years, a clinical diagnosis of NSTE-ACS according to ESC Guidelines [15,16] and a scheduled coronary angiography.

Major exclusion criteria were (1) prior myocardial infarction, percutaneous coronary intervention or cardiac surgery; (2) significant valvular dysfunction; (3) left bundle branch block or atrial fibrillation; (4) inadequate image quality; and (5) a final diagnosis other than NSTE-ACS. The latter included myocarditis, Takotsubo syndrome, pulmonary embolism, or other forms of myocardial infarction with non-obstructive coronary arteries (MINOCA). Patients with very high-risk features requiring immediate angiography (<2 h) were also excluded.

**Figure 1:**
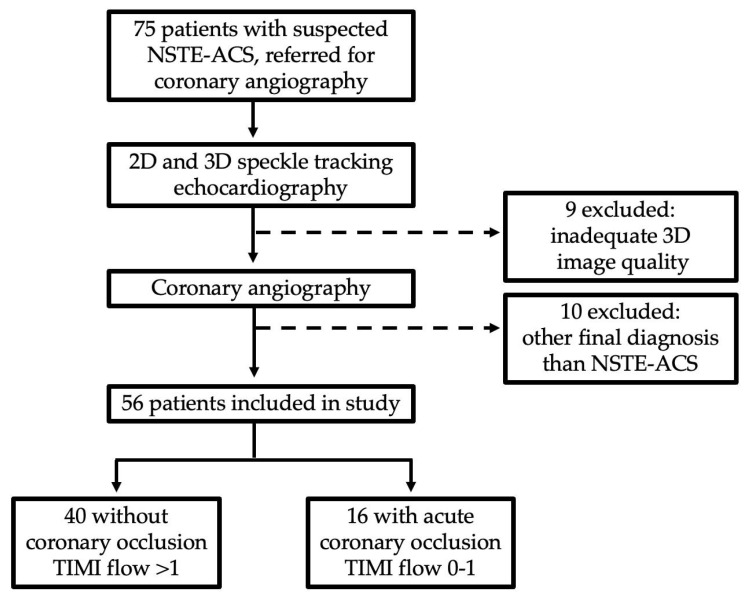
Flowchart of patient selection.

A total of 75 patients underwent echocardiography. Of these, 10 patients were excluded due to a different final diagnosis, whereas 9 were excluded due to an inadequate image quality of 3D echocardiography. The final study population comprised 56 patients (Figure 1).

ECGs were evaluated at admission and were described as ischemic if ST depression or T-wave changes were present. Biochemical analyses including cardiac troponins were collected from the index hospital and the invasive center. Echocardiography was performed prior to coronary angiography. Retrospectively, patients were grouped according to whether the culprit lesion was occluded or non-occluded. All patients received medical treatment according to guidelines [15,16].

Written informed consent was obtained from all patients, and the study was performed according to the second Helsinki declaration and was approved by Regional Committee for Medical Research Ethics (REK 2013/188).

### 2.2. Echocardiography

Echocardiography was performed with the Artida scanner (Toshiba/Canon Medical Systems, Tokyo, Japan). Conventional 2D grayscale loops were obtained from the apical views (four-chamber, two-chamber, and long-axis views) and three parasternal short axis views (mitral valve, papillary muscle and apex). Mean frame rate was 55.4 ± 10.0 for 2D grayscale imaging. 3D imaging was performed from an apical position, and a pyramidal volume consisting of 4–6 sub-volumes was acquired over 4–6 consecutive cardiac cycles during a single breath-hold, using a 3D matrix array 2.5 MHz transducer. Mean frame rate was 22.6 ± 1.8 for 3D images. The echocardiographic data were analyzed off-line blinded to clinical information.

### 2.3. Speckle Tracking Echocardiography

Myocardial strain was assessed using 2D and 3D STE with a vendor specific wall motion tracking software (UltraExtend with Advanced Cardiology Package v. 2.7, Toshiba/Canon Medical Systems, Tokyo, Japan).

For 2D STE, longitudinal strain was measured from three apical views and circumferential strain from 2–3 short-axis views. Endocardial borders were outlined at end-systole, adjusting the region of interest to match myocardial wall thickness. The software subsequently performed frame-by-frame speckle tracking. Segments that failed to track were manually corrected, and if tracking issues persisted in more than two segments per view, the analysis was excluded. Separate strain values for endocardial, mid-myocardial, and epicardial layers were provided, and endocardial strain was used for further analysis. Strain values from 16 segments were averaged to global longitudinal strain (2D GLS) and global circumferential strain (2D GCS). However, valid 2D GCS was not a mandatory criterion for inclusion.

In 3D STE, datasets were displayed in a five-plane view. After placing three landmarks in 2 orthogonal apical views, the software automatically identified the LV endocardial and epicardial borders, allowing for manual adjustments. The speckle-tracking analysis provided longitudinal and circumferential strain, along with LV volumes and EF. Analyses were excluded if more than three segments failed tracking despite manual correction. Global longitudinal strain (3D GLS) and global circumferential strain (3D GCS) were calculated by averaging peak systolic strain values from 16 LV segments.

### 2.4. Coronary Angiography

All patients underwent coronary angiography, performed on clinical indication, using digital image acquisition and storage. Acute coronary occlusion was defined as TIMI (Thrombolysis in Myocardial Infarction) flow grade 0 or 1 in the presumed culprit artery, based on angiographic appearance and overall clinical presentation, including ECG findings. In cases with multiple severe lesions, the culprit vessel was determined by consensus. Chronic total occlusions were identified based on typical angiographic features and clinical context and were not classified as acute occlusions. Percutaneous coronary intervention (PCI) was performed when judged appropriate given angiographic and clinical data.

### 2.5. Statistical Analyses

Continuous variables are presented as mean ± SD or median (interquartile range), and differences between groups were analyzed with Student’s *t*-test or Mann–Whitney U-test, as appropriate. Categorical variables are presented as numbers and percentages, and differences between groups were analyzed by Chi square test or Fisher’s exact test. Feasibility of 2D and 3D STE was compared using McNemar’s test for paired proportions. Correlations between the number of significant coronary stenoses and echocardiographic parameters were assessed using Spearman’s rank correlation coefficient (rho). The diagnostic accuracy of the echocardiographic parameters was evaluated with receiver operating characteristic (ROC) curve analysis. Optimal cut-off values were defined using Youden’s index. Area under the curve (AUC), sensitivity, specificity and positive and negative predictive values (PPV and NPV) for detection of acute coronary occlusion were calculated.

Univariate logistic regression analyses were performed to identify clinical and echocardiographic variables associated with acute coronary artery occlusion. Variables with *p* < 0.05 in univariate analysis, as well as clinically relevant parameters, were considered for further modeling. To reduce the risk of overfitting given the limited number of outcome events, multivariate logistic regression models were limited to a maximum of two covariates. Specifically, we constructed three separate models, each including 3D GLS in combination with one additional variable: age (Model 1), troponin T (Model 2), or 2D GLS (Model 3). This approach allowed us to assess the independent predictive value of 3D GLS while adjusting for selected clinical or echocardiographic covariates.

Multicollinearity was evaluated using variance inflation factors (VIF), with VIF > 5 or pairwise correlation coefficients > 0.7 considered indicative of collinearity [17]. Odds ratios (ORs) with 95% confidence intervals (CIs) were reported for all models.

Interobserver and intraobserver variability for 3D STE was assessed by reanalyzing 10 randomly selected patients and reported as intra-class correlation coefficients.

ROC analyses were performed using GraphPad Prism v. 10, GraphPad Software, Boston, MA, USA, while all other statistical analyses were performed on IBM SPSS Statistics v. 30 (IBM Corp, Armonk, NY, USA). A two-tailed *p*-value of <0.05 was considered statistically significant.

## 3. Results

### 3.1. Demographic and Angiographic Data

We included 56 patients with a mean age of 64.0 ± 10.8 years, in whom 45 (80%) were males. The median time from index admission at the local hospital to coronary angiography was 2 (IQR 1-2) days. Of the total cohort, 50 patients (89%) fulfilled diagnostic criteria for NSTEMI, while 6 patients (11%) were classified as UA. Sixteen patients (29%) had acute coronary occlusion as assessed by coronary angiography. Table 1 summarizes clinical characteristics of patients with and without occlusion. There were no differences in age, gender, or cardiovascular risk factors between the groups.

Details of the angiographic findings and revascularization therapy are showed in Table 2. The left anterior descending (LAD) was occluded in seven cases (44%) and the right coronary artery (RCA) in six cases (38%), while the left circumflex artery (CX) was occluded in three cases (19%). Three-vessel disease was more common in patients with occlusion (*p* = 0.006).

### 3.2. Echocardiographic Findings

**Figure 2:**
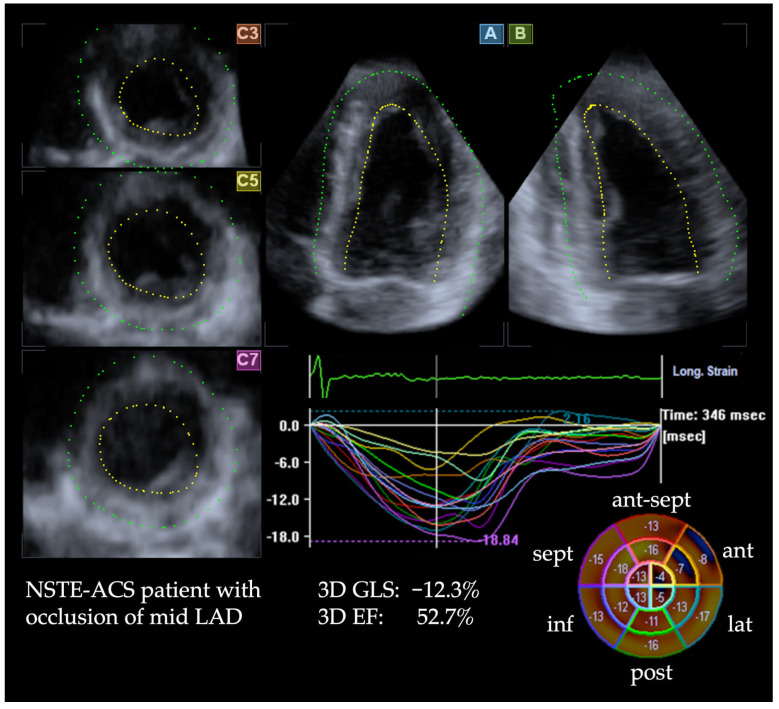
3D speckle tracking echocardiographic strain analysis of a patient with an occlusion of the mid segment of the left anterior descending (LAD) coronary artery. The apical views (A and B) and corresponding short-axis slices (C3, C5, C7) are shown along with segmental strain curves and a bull’s-eye plot. While 3D ejection fraction (EF) was normal (52.7%), 3D global longitudinal strain (GLS) was significantly reduced (−12.3%). The bull’s-eye plot in the lower right illustrates markedly reduced regional longitudinal strain in the anterior segments, typically perfused by the LAD.

Patients with acute coronary occlusion had similar 3D EF compared to patients without occlusion (52.5% ± 4.7 vs. 54.7% ± 4.7, *p* = 0.16). In contrast, GLS and GCS by both 2D 3D STE were significantly lower (i.e., more impaired) in the occlusion group (all *p* < 0.05), (Table 3). Figure 2 illustrates a 3D STE analysis of a patient with an acute occlusion, in which 3D EF was normal (52.7%), while 3D GLS was reduced (−12.3%).

Receiver operating characteristic (ROC) analyses showed that 3D GLS had the highest discriminative ability for identifying acute coronary occlusion (AUC 0.81), followed by 2D GLS (AUC 0.78). Optimal cut-off values were −14.4% for 3D GLS and −14.8% for 2D GLS, both yielding a sensitivity of 75%, with a specificity of 78% and 73%, respectively.

**Figure 3:**
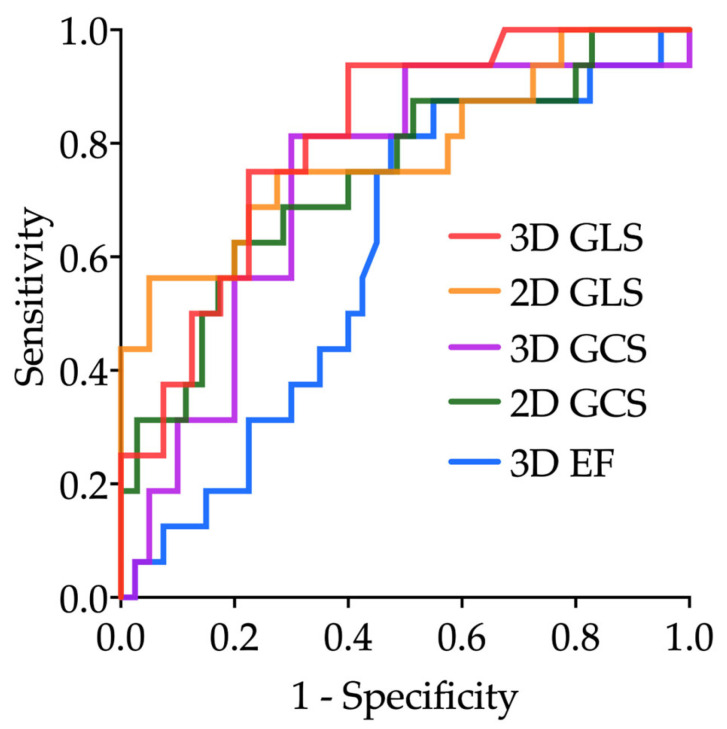
Receiver operating characteristic (ROC) curves for echocardiographic parameters in detecting acute coronary artery occlusion in patients with NSTE-ACS.

Both 3D GCS and 2D GCS demonstrated moderate accuracy (AUCs 0.73 and 0.74), while 3D EF had lower diagnostic performance (AUC 0.61). Detailed diagnostic characteristics are provided in Figure 3 and Table 4.

### 3.3. Predictors of Coronary Occlusion

In univariate logistic regression, reduced 3D GLS was significantly associated with acute coronary occlusion (OR = 1.71 per 1% absolute reduction; 95% CI: 1.24–2.36; *p* = 0.001), along with 2D GLS (OR = 1.73; 95% CI: 1.25–2.38; *p* = 0.001), 2D GCS (OR = 1.20; 95% CI: 1.05–1.37; *p* = 0.006), and 3D GCS (OR = 1.18; 95% CI: 1.02–1.37; *p* = 0.03). In contrast, 3D EF was not significantly associated (OR = 0.67 per 5% increase; 95% CI: 0.38–1.18; *p* = 0.16), nor were clinical variables such as age, hypercholesterolemia, hypertension, ischemic ECG changes, or troponin T.

In multivariate models, 3D GLS remained significantly associated with occlusion when adjusted for age or troponin T, with identical effect estimates (OR = 1.74, 95% CI: 1.24–2.42; *p* = 0.001). In a combined model including both 3D and 2D GLS, neither variable reached statistical significance (3D GLS: *p* = 0.06; 2D GLS: *p* = 0.05), although both showed independent trends toward association with occlusion (Table 5).

### 3.4. Association Between Coronary Disease Severity and Echocardiographic Parameters

To further explore the relationship between echocardiographic parameters and coronary disease burden, we compared 3D and 2D strain and 3D EF values in patients with (*n* = 47) and without (*n* = 9) at least one significant coronary stenosis (>70% luminal narrowing). Both 3D GLS and 2D GLS were significantly lower in patients with significant stenosis compared to those without (3D GLS: −14.3 ± 2.5% vs. −16.8 ± 2.3%, *p* = 0.005; 2D GLS: −14.4 ± 2.5% vs. −16.9 ± 2.1%, *p* = 0.012). No significant differences were observed for 3D EF, 3D GCS or 2D GCS between the two groups (Table 6).

Correlation analyses further demonstrated a weak but significant correlation between the number of significant stenoses per patient and both 3D GLS (Spearman’s rho = 0.27, *p* = 0.045) and 2D GLS (rho = 0.31, *p* = 0.02), indicating that worsening strain is associated with increasing coronary disease burden. No significant correlations were found between the number of significant stenoses and 3D EF, 3D GCS, or 2D GCS.

### 3.5. Feasibility and Reproducibility

Among the 65 initially examined patients, 9 (14%) were excluded due to inadequate image quality for assessment with 3D STE. In contrast, only three patients (5%) were unfeasible for 2D longitudinal strain (*p* = 0.03 vs. 3D STE) and five (8%) for 2D circumferential strain (*p* = 0.42 vs. 3D STE).

In the 56 patients included in the study, 3D longitudinal strain was assessable in 847/896 (95%) LV segments, and 3D circumferential strain in 856/896 (96%) segments. In comparison, 2D longitudinal strain was assessable in 894/1008 (89%) LV segments and 2D circumferential strain in 614/690 (89%) segments. Thus, the proportion of segments analyzed were higher for 3D STE than 2D STE (both *p* < 0.001).

Intraobserver and interobserver intraclass correlations were performed in 10 patients and were 0.87 and 0.81, respectively, for 3D GLS and 0.93 and 0.92 for 3D GCS measurements. For 2D STE, we have performed intraobserver and interobserver variability analysis in a previous study [18].

## 4. Discussion

### 4.1. Summary of Findings

In this prospective study of NSTE-ACS patients, we evaluated the diagnostic performance of 3D and 2D STE for identifying acute coronary occlusion. Both 3D and 2D GLS showed the highest accuracy, with AUC values of 0.81 and 0.78, respectively. 3D and 2D GCS demonstrated intermediate discriminative ability (AUC 0.73 and 0.74), while 3D EF had the lowest overall accuracy (AUC 0.61). These findings underscore the diagnostic value of GLS compared to EF in this setting, with GCS offering moderate, though less robust, discriminative contribution.

In addition, both 3D and 2D GLS correlated with the extent of coronary artery disease, indicating that strain parameters reflect not only the presence of occlusion but also overall ischemic burden.

However, 3D STE did not provide incremental diagnostic value over 2D STE, and its feasibility was lower—particularly in comparison to 2D longitudinal strain.

### 4.2. Comparison with Previous Studies

Few studies have directly examined the diagnostic role of 3D STE in the setting of NSTE-ACS. Biswas et al. investigated 3D strain parameters in patients with NSTE-ACS and found that both 3D GLS and 3D GCS were significantly reduced in patients with severe coronary stenosis (>70% luminal narrowing) [12]. A 3D GLS threshold of −13.5% yielded an AUC of 0.84, with 89% sensitivity and 71% specificity for identifying significant stenosis. They also reported more impaired GLS in patients with occlusion compared to those without (−9.8 ± 3.0% vs. −13.0 ± 2.8%), although no ROC analysis was provided for this subgroup.

Our study extends these findings by evaluating the ability of 3D STE specifically for identifying acute coronary occlusion. We found that a 3D GLS cut-off of −14.4% provided an AUC of 0.81, with 75% sensitivity and 78% specificity.

Importantly, the study by Biswas did not include 2D STE, preventing a direct comparison between modalities. By incorporating both 2D and 3D STE, our study provides a more comprehensive assessment of their relative performance in detecting occlusion.

Similarly to Biswas et al. [12], our results also showed that both 2D and 3D GLS were significantly associated with the extent of coronary artery disease. Despite discrepancies in absolute strain values—likely attributable to vendor-specific algorithms and cohort composition—both studies underscore the sensitivity of longitudinal strain to the overall extent of myocardial ischemia, beyond simply detecting occlusion.

Other studies have explored 3D STE in relation to coronary complexity among NSTE-ACS patients. Cai et al. and Raslan et al. both demonstrated lower 3D GLS in patients with high SYNTAX scores, also indicating an association between 3D strain and global coronary disease burden [13,14].

Beyond 3D STE, prior studies have validated 2D strain imaging for detecting myocardial ischemia. Grenne et al. and Eek et al. demonstrated that territorial and regional strain indices could accurately identify acute coronary occlusions in NSTE-ACS [6,7]. Supporting its broader diagnostic potential in stable CAD, Gaibazzi et al. showed that resting 2D GLS could detect significant coronary artery stenosis (>50% luminal narrowing) with accuracy comparable to stress-derived indices [8]. Similarly, a recent study by Koulaouzidis et al. reported that reduced 2D GLS could identify significant CAD in patients with preserved EF and no regional wall motion abnormalities, with diagnostic accuracy increasing in proportion to the extent of disease [19].

Taken together, these studies establish 2D STE as a sensitive tool for detecting subclinical myocardial dysfunction. Our results reinforce these findings and support the continued clinical utility of 2D STE in NSTE-ACS while also demonstrating that 3D STE may serve as a complementary modality where feasible.

### 4.3. Feasibility and Future Directions for 3D STE

While 3D STE holds promise for streamlining strain assessment by enabling a full-volume acquisition and simultaneous analysis of multiple parameters, feasibility remains a key limitation. In our study, 14% patients were excluded due to inadequate 3D image quality—considerably higher than for 2D longitudinal strain, where feasibility was 95%.

Limitations of 3D STE include lower spatial and temporal resolution and greater dependence on optimal image quality. Multi-beat acquisitions may also introduce stitching artifacts that compromise tracking accuracy. Even more than in 2D techniques, 3D STE is affected by inter-vendor variability and a lack of standardization, further complicating its clinical implementation [5,9].

Nonetheless, 3D STE addresses several inherent weaknesses of 2D strain imaging, including susceptibility to foreshortened views and out-of-plane speckle motion. It also enables more time-efficient analysis by capturing the entire left ventricle in a single acquisition. Moreover, 3D STE allows for a comprehensive assessment of additional deformation parameters such as twist, torsion, and shear strain, which may offer further diagnostic or prognostic value—though these measures remain largely investigational [9].

Looking ahead, improvements in transducer design, real-time 3D imaging, and AI-based automation may enhance image quality, reduce variability, and increase the reliability of tracking algorithms. With these advancements, the clinical feasibility and diagnostic utility of 3D STE may improve substantially. Until then, 2D STE remains the most accessible and reliable modality for myocardial deformation imaging in acute coronary syndromes [5].

### 4.4. Clinical Implications

Early identification of acute coronary occlusion in NSTE-ACS is critical, given its association with worse outcomes and the potential benefit of early invasive management. Traditional diagnostic tools, such as ECG and wall motion analysis, often fail to detect subtle myocardial injury in these patients. Our findings suggest that both 2D and 3D GLS can support early risk stratification and may inform decisions regarding timely coronary angiography. While 3D STE may evolve into a valuable complementary technique, 2D STE remains the preferred modality in clinical practice due to its higher feasibility and broader availability.

### 4.5. Study Limitations

This study has several limitations. First, the sample size was relatively small, and the number of patients with acute coronary occlusion was low, which limited statistical power—particularly for subgroup analyses and multivariable modeling.

Second, patients were recruited at a single tertiary center using convenience sampling. Several patient groups were also excluded based on predefined criteria, including prior myocardial infarction or revascularization, very high-risk presentations requiring immediate angiography, and final diagnoses other than NSTE-ACS. This increases the risk of selection bias and limits generalizability to broader populations with acute chest pain.

Third, 3D strain analysis showed only moderate feasibility, as a substantial number of otherwise eligible patients were excluded due to inadequate image quality.

Fourth, echocardiography was performed 1–3 days after index admission rather than acutely.

Finally, as all patients received guideline-based therapy prior to imaging, this may have led to spontaneous thrombus resolution and partial recovery of myocardial function.

## 5. Conclusions

In this prospective study of NSTE-ACS patients, 2D and 3D GLS showed higher diagnostic accuracy than 3D EF in identifying acute coronary occlusion and demonstrated a modest correlation with overall coronary disease burden. However, 3D STE did not offer added diagnostic value over 2D STE and had lower feasibility.

Given the limited sample size and exclusion of several patient groups, the findings should be interpreted with caution. While 2D STE remains the more established modality in current practice, 3D STE may play a complementary role as technology advances.

## Figures and Tables

**Table 1:** Patient characteristics.

	No Occlusion TIMI > 1 *n* = 40	Occlusion TIMI 0–1 *n* = 16	Total *N* = 56	*p*-Value
**Demographics**				
Age, years	64.0 ± 10.7	63.9 ± 11.2	64.0 ± 10.8	0.98
Male	32 (80%)	13 (81%)	45 (80%)	1.00
BMI, kg/m^2^	26.1 ± 3.7	25.9 ± 3.6	26.0 ± 3.6	0.90
Systolic BP, mmHg	140 ± 23	140 ± 22	140 ± 22	0.94
Diastolic BP, mmHg	78 ± 14	76 ± 12	77 ± 13	0.68
Heart rate, bpm	68 ± 10	66 ± 8	67 ± 10	0.53
**Risk factors**				
Smoking	13 (33%)	5 (31%)	18 (32%)	0.93
Hypercholesterolemia	18 (45%)	8 (50%)	26 (54%)	0.74
Hypertension	13 (33%)	6 (38%)	19 (34%)	0.72
Diabetes mellitus	5 (13%)	3 (19)	8 (14%)	0.68
Family history of CAD *	19 (48%)	8 (50%)	27 (48%)	0.87
Ischemic ECG	23 (58%)	11 (69%)	34 (61%)	0.44
**Biomarkers**				
Troponin T, ng/L	309 (79–1017)	362 (196–821)	332 (128–987)	0.25
Cholesterol, mmol/l	5.3 (4.6–6.6)	5.3 (4.9–6.2)	5.3 (4.7–6.3)	0.95
**Final diagnosis**				
Unstable angina	6 (15%)	0 (0%)	6 (11%)	0.17
NSTEMI	34 (85%)	16 (100%)	50 (89%)	0.17

Values are mean ± SD, *n* (%) or median (interquartile range). * CAD in first-degree relative prior to age 55 (men) or 65 (women). BMI = body mass index; BP = blood pressure; CAD = coronary artery disease; ECG = electrocardiogram; NSTEMI = non ST-elevation myocardial infarction; TIMI = thrombolysis in myocardial infarction flow grade.

**Table 2:** Angiographic findings and revascularization therapy.

	No Occlusion TIMI > 1 *n* = 40	Occlusion TIMI 0–1 *n* = 16	Total *N* = 56	*p*-Value
**Angiographic findings**				
No significant CAD	9 (23%)	0 (0%)	9 (16%)	0.048
Significant CAD	31 (77%)	16 (100%)	47 (84%)	0.048
**CAD burden**				
1-vessel disease	21 (53%)	10 (63%)	31 (55%)	0.50
2-vessel disease	9 (23%)	1 (6%)	10 (18%)	0.25
3-vessel disease	1 (3%)	5 (31%)	6 (11%)	0.006
**Revascularization**				
PCI	29 (73%)	13 (81%)	42 (75%)	0.73
CABG	2 (5%)	3 (19%)	5 (9%)	0.14
No intervention	9 (23%)	0 (0%)	9 (16%)	0.048

Values are *n* (%). CABG = coronary artery bypass grafting; CAD = coronary artery disease; PCI = percutaneous coronary intervention; TIMI = thrombolysis in myocardial infarction flow grade.

**Table 3:** Echocardiographic findings.

	No Occlusion TIMI > 1 *n* = 40	Occlusion TIMI 0–1 *n* = 16	Total *N* = 56	*p*-Value
3D GLS (%)	−15.5 ± 2.1	−12.5 ± 2.7	−14.7 ± 2.6	<0.001
2D GLS (%)	−15.6 ± 2.0	−12.6 ± 2.8	−14.7 ± 2.6	<0.001
3D GCS (%)	−27.8 ± 4.3	−24.8 ± 4.4	−26.9 ± 4.5	0.02
2D GCS (%)	−22.9 ± 4.7	−18.1 ± 5.5	−21.4 ± 5.4	0.002
3D EF (%)	54.7 ± 5.7	52.5 ± 4.7	54.1 ± 5.5	0.16

Values are mean ± SD. 2D = 2-dimensional; 3D = 3-dimensional; EF = ejection fraction; GCS = global circumferential strain; GLS = global longitudinal strain, TIMI = thrombolysis in myocardial infarction flow grade.

**Table 4:** Diagnostic accuracy of echocardiographic parameters for identifying acute coronary artery occlusion, based on ROC analysis (see Figure 3 for corresponding curves).

	AUC (95% CI)	Optimal Cut-Off	Sensitivity	Specificity	PPV	NPV
3D GLS	0.81 (0.69–0.93)	−14.4%	75%	78%	58%	89%
2D GLS	0.78 (0.64–0.93)	−14.8%	75%	73%	53%	88%
3D GCS	0.73 (0.58–0.88)	−26.0%	75%	70%	50%	88%
2D GCS	0.74 (0.59–0.89)	−22.3%	75%	60%	43%	86%
3D EF	0.61 (0.45–0.76)	53.8%	75%	55%	40%	85%

2D = 2-dimensional; 3D = 3-dimensional; AUC = area under curve; CI = confidence interval; EF = ejection fraction; GCS = global circumferential strain; GLS = global longitudinal strain; PPV = positive predictive value; NPV = negative predictive value.

**Table 5:** Multivariate logistic regression models for prediction of acute coronary occlusion.

	Model 1: 3D GLS + Age		Model 2: 3D GLS + Troponin T		Model 3: 3D GLS + 2D GLS ^†^	
	OR (95% CI)	*p*	OR (95% CI)	*p*	OR (95% CI)	*p*
Age (per 5 years)	1.08 (0.78–1.47)	0.64				
Troponin T (per 100 ng/L)			1.00 (0.90–1.11)	0.93		
2D GLS					1.43 (1.00–2.06)	0.05
3D GLS	1.74 (1.24–2.42)	0.001	1.74 (1.24–2.42)	0.001	1.42 (0.99–2.06)	0.06

2D = 2-dimensional; 3D = 3-dimensional; CI = confidence interval; GCS = global circumferential strain; GLS = global longitudinal strain; OR = odds ratio. ^†^ No significant multicollinearity was observed between 3D GLS and 2D GLS (Pearson r = 0.65, VIF = 1.72). Variables were scaled as follows: age per 5-year increase, 3D GLS and 2D GLS per 1% absolute reduction, and troponin T per 100 ng/L increase.

**Table 6:** Echocardiographic parameters for detection of significant CAD.

	No Significant CAD *n* = 9	Significant CAD *n* = 47	Total *N* = 56	*p*-Value
3D GLS (%)	−16.8 ± 2.3	−14.3 ± 2.5	−14.7 ± 2.6	0.005
2D GLS (%)	−16.9 ± 2.1	−14.4 ± 2.5	−14.7 ± 2.6	0.012
3D GCS (%)	−26.7 ± 3.8	−26.9 ± 4.6	−26.9 ± 4.5	0.99
2D GCS (%)	−23.9 ± 3.8	−20.9 ± 5.5	−21.4 ± 5.4	0.13
3D EF (%)	55.3 ± 5.0	53.8 ± 5.6	54.1 ± 5.5	0.62

Values are mean ± SD. 2D = 2-dimensional; 3D = 3-dimensional; CAD = coronary artery disease; EF = ejection fraction; GCS = global circumferential strain; GLS = global longitudinal strain.

## Data Availability

The data presented in this study are available on request from the corresponding author.

## Abbreviations

The following abbreviations are used in this manuscript:

2D	Two-dimensional
3D	Three-dimensional
ACS	Acute coronary syndrome
CAD	Coronary artery disease
CX	Circumflex coronary artery
EF	Ejection fraction
GCS	Global circumferential strain
GLS	Global longitudinal strain
LAD	Left anterior descending coronary artery
LV	Left ventricle/left ventricular
NSTE-ACS	Non-ST segment elevation acute coronary syndrome
PCI	Percutaneous coronary intervention
RCA	Right coronary artery
STE	Speckle tracking echocardiography
STEMI	ST segment elevation myocardial infarction
UA	Unstable angina
